# Effect of Electrical Heat Carrier Temperature on Bacterial Leakage of Endodontically Treated Teeth Using a Bioceramic Sealer

**DOI:** 10.1002/cre2.70059

**Published:** 2025-02-03

**Authors:** Mir Ahmad Nabavi, Mahmood Reza Kalantar Motamedi, Pedram Fattahi, Saber Khazaei

**Affiliations:** ^1^ School of Dentistry Kermanshah University of Medical Sciences Kermanshah Iran; ^2^ Dental Research Center, Dental Research Institute, School of Dentistry Isfahan University of Medical Sciences Isfahan Iran; ^3^ Student Research Centre Qazvin University of Medical Sciences Qazvin Iran; ^4^ Department of Endodontics, School of Dentistry Kermanshah University of Medical Sciences Kermanshah Iran

**Keywords:** bacterial leakage, bioceramic sealer, root canal filling materials, root canal obturation

## Abstract

**Objectives:**

The aim of this study is to investigate the effect of electrical heat carrier temperature on bacterial leakage of root canals obturated with the continuous wave of condensation (CWC) technique and a bioceramic sealer.

**Methods:**

This ex vivo experimental study was conducted on 92 extracted single‐rooted teeth. The teeth were subjected to endodontic treatment and obturated with the Endoseal MTA bioceramic sealer by the CWC technique using two different temperature settings of the electrical heat carrier: 120°C (group G120, *n* = 41) and 200°C (group G200, *n* = 41). A positive and a negative control group were also considered (*n* = 5 each). Bacterial leakage was assessed over a 40‐day period using a bacterial leakage model. The incidence of bacterial leakage was compared between the experimental groups using the log‐rank test. The significance level was set at 0.05.

**Results:**

The median survival rate was 39.0 (25.0) days in the G120 group and 34.0 (25.0) days in the G200 group. Despite a slightly higher survival rate in the G120 group, the difference between the two experimental groups was not statistically significant (*p* = 0.612).

**Conclusion:**

The tested temperatures of the electrical heat carrier (120°C and 200°C) did not have a significant effect on bacterial leakage of root canals obturated by the CWC technique and the Endoseal MTA bioceramic sealer.

## 1 Introduction

Coronal leakage is among the important causes of endodontic treatment failure (Saunders and Saunders 1994). It is necessary to minimize leakage (Seltzer and Bender 1965) and seal the communication paths between the root canal system and the periapical tissue to prevent bacterial invasion (Kakehashi, Stanley, and Fitzgerald 1965). Microleakage may occur through the dentin–sealer or sealer–gutta–percha interface, or within the sealer itself (Malone and Donnelly 1997; Wu et al. 1993). The gaps present in the root filling can serve as a reservoir for microorganisms and lead to periapical disease and subsequent treatment failure (Van der Sluis, Wu, and Wesselink 2005).

Bioceramic sealers have gained increasing popularity in recent years, and many studies have compared their properties with other sealer types (Silva Almeida et al. 2017; Camilleri 2017; Guivarc'h et al. 2020). Optimal dimensional stability and even volume expansion during the setting reaction are among the desirable properties of bioceramic sealers (Silva Almeida et al. 2017; Zhou et al. 2013), which can theoretically enhance the sealing ability. Additionally, the formation of mineral layers during the setting process creates a chemical bond to the dentin walls in the biological environment, which can improve the sealing ability of such sealers (Silva Almeida et al. 2017; Camilleri 2017; Lim et al. 2020).

Various techniques may be adopted for root canal obturation. The continuous wave of condensation (CWC) is one such technique, which was introduced by Buchanan (Buchanan 1994). This technique involves use of the warm gutta–percha technique to create a uniform mass that can flow and fill the lateral canals, grooves, and depressions of the canal space more efficiently than the lateral compaction technique (DuLac et al. 1999; Goldberg, Artaza, and De Silvio 2001). Moreover, the CWC technique can create a more hermetic seal against coronal microbial leakage in comparison to the lateral compaction technique (Bowman and Baumgartner 2002).

In the CWC technique, the penetration depth of the electrical heat carrier is 5–7 mm from the apex with the suggested temperature of 200°C (Buchanan 1994). However, such a high temperature may lead to chemical or physical changes (Viapiana et al. 2015). The use of heat may affect the setting time, film thickness, bond strength, and flow rate of some sealer types (Heran et al. 2019; Camilleri 2015). However, it has been shown that heat does not affect the quality of root filling when using the EndoSequence BC sealer (Celikten et al. 2015; McMichael, Primus, and Opperman 2016). Hence, it is crucial to take into account the type of sealer while choosing the obturation technique (Camilleri 2015).

The sealing ability of sealers may also be adversely affected by the changes in sealer properties caused by heat in the CWC technique, which may subsequently lead to bacterial leakage. To overcome this drawback, Abdellatif et al. suggested a modified technique by using a lower heat carrier temperature (Abdellatif et al. 2021). However, to the best of the authors’ knowledge, no previous study is available on this modification, addressing the leakage of root filling.

Hence, the purpose of the present study was to investigate the effect of electrical heat carrier temperature on bacterial leakage of root canals obturated with the Endoseal MTA bioceramic sealer using the CWC technique.

## 2 Materials and Methods

### 2.1 Study Design and Sample Selection

Based on pre‐test data, with 80% power of the test and a 90% confidence interval, the required sample size was 41 cases in each experimental group (i.e., 82 cases in total). Additionally, five cases were allocated for each of the positive and negative control groups. In total, this ex vivo experimental study was performed on 92 single‐rooted teeth extracted for orthodontic or periodontal reasons.

The included teeth had almost straight roots (Schneider 1971) with mature apices and no fracture, calcification, cracks, caries, and internal or external root resorption defects. The protocol of the study was approved by the regional bioethics committee of the Kermanshah University of Medical Sciences (#IR.KUMS.REC.1400.837).

### 2.2 Sample Preparation

**Figure 1:**
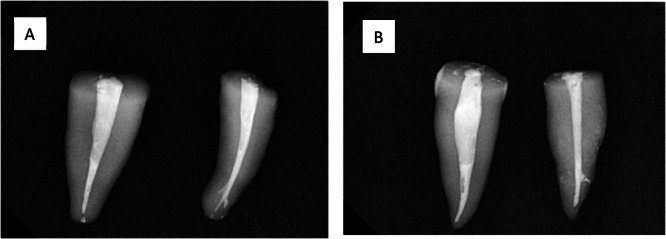
Radiographs of the obturated teeth: (A) samples processed at 200°C and (B) samples processed at 120°C.

The teeth were cleaned from periodontal tissue residues and were disinfected by immersion in 5.25% sodium hypochlorite for 10 min. The teeth were then decoronated by one of the authors (M. N.) using a fissure bur to equalize the root length in all teeth. Subsequently, another author (S. Kh.) re‐inspected the roots to ensure that they were similar in length. After decoronation, the cross‐section of the root canals was examined to determine if they were mostly round. A root canal was considered mostly round and included in the study if the ratio of the long to short diameter was less than 2 (Kalantar Motamedi et al. 2021). Next, a #10 K‐file (Mani Inc. Tochigi, Japan) was inserted into the canal until the tip of the file was visible. The working length was determined by subtracting 1 mm from the file length. A glide path was established by using a #15 K‐file (Mani). Afterward, the root canals were instrumented up to the F3 (30/.08) rotary file (Denco, Shenzhen, China). The root canals were irrigated with 5.25% sodium hypochlorite after using each file. Following cleaning and shaping of the root canals, the smear layer was removed with 17% EDTA and 5.25% sodium hypochlorite, and the canals were rinsed with saline. The canals were subsequently dried with sterile paper points (Meta Biomed, Cheongju, South Korea). The teeth were then randomly divided into two experimental groups (*n* = 41 each) and two positive and negative control groups (*n* = 5 each). In the first experimental group (G120), the root canals were filled with the Endoseal MTA sealer (Maruchi, Wonju, South Korea) using the injection technique as suggested by the manufacturer, and then the master gutta–percha point (Meta Biomed) (35/.04 or 40/.04 depending on the apical constriction and the presence of tug‐back) was gently placed on the working length. If there was no apical tug‐back, the tooth was excluded from the study. The temperature of the Fast‐Pack electrical heat carrier (Eighteeth, Changzhou, China) was set at 120°C, and the gutta–percha points were cut at 5 mm from the apex and packed with a plugger. In the next step, the remaining root canal space was back‐filled using Fast‐Fill (Eighteeth), and the temperature of the melting gutta–percha was set at 180°C. The gutta–percha was packed with a hand plugger to the level of the orifice. In the second experimental group (G200), the same procedures were performed as those for the first group, but the master gutta–percha point was cut using a temperature of 200°C. In both the experimental groups, radiographs were taken to confirm the optimal quality of obturation (Figure 1).

The negative control samples were also cleaned and shaped, and obturated with the cold lateral compaction technique using the Endoseal MTA sealer (Williamson et al. 2009). The root canals in the positive control samples were left empty after cleaning and shaping. The root surfaces in both the experimental and positive control groups were coated with two layers of nail varnish, except for the 2 mm apical region. In the negative control group, all surfaces were completely coated with two layers of nail varnish. All the samples were stored at 37°C and 100% humidity for 48 h (Milani et al. 2019).

### 2.3 Bacterial Leakage Testing

The double‐chamber Eppendorf method was adopted to quantify bacterial leakage. The required apparatus was constructed using two 1.5 mL microtubes. The samples were placed in the microtubes and then the ends of the microtubes were cut with a #20 blade such that 2–3 mm of the root tip was exposed. The gap between the tooth and the microtube was sealed with cyanoacrylate glue. The samples were sterilized with ethylene oxide gas for 12 h. Under aseptic conditions and a Class II biological hood, Brain–heart infusion broth culture medium (Merck, Darmstadt, Germany) was added to 1.5 mL microtubes using a sterile sampler, and then the tooth–microtube assembly was placed in it such that 2–3 mm of the tooth apex was dipped in the culture medium. To ensure no contamination, the samples were incubated at 37°C for 72 h. Turbidity of the culture medium would indicate contamination, and such samples would be excluded from the study.

A bacterial suspension of Enterococcus faecalis (ATCC 1858; E. faecalis) with 0.5 McFarland standard concentration was then prepared. Every 24 h, 0.1 mL of the bacterial suspension was added to the upper chamber of the teeth present in the microtube. The entire assembly was incubated at 37°C for 40 days (61). The samples were evaluated on a daily basis. The turbidity of the brain–heart infusion culture medium indicated bacterial growth, and the time (day) of turbidity occurrence was recorded. To confirm that the contaminating microorganism was E. faecalis, a sample was taken from the bottom of the microtube and cultured on bile esculin agar medium (Himedia; Mombia, India) and incubated at 37°C. Grown colonies would cause a color shift to black, which would indicate the presence of E. faecalis in the contaminated sample.

### 2.4 Statistical Analysis

The measures of central tendency and dispersion were reported using numbers and proportions. The median survival rate was also calculated for each group. The proportion of bacterial colonization was compared between the groups using Fisher's exact test. The Kaplan–Meier survival analysis was performed, and a log‐rank test was utilized to compare the study groups using SPSS version 18 (SPSS Inc. Chicago, USA). The significance level was set at 0.05. In this study, the null hypothesis was that there is no significant difference in bacterial leakage between root canals obturated with an electrical heat carrier at 120°C and those at 200°C.

## 3 Results

**Figure 2:**
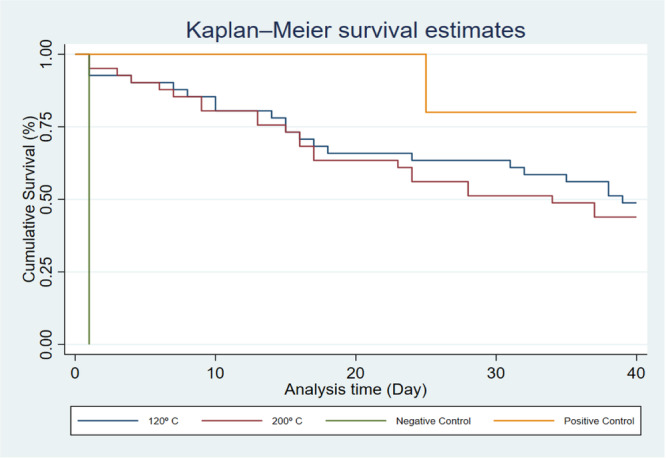
Kaplan–Meier estimate to calculate the cumulative survival rate.

All five samples in the positive control group showed bacterial growth on day one. One out of five samples in the negative control group showed bacterial growth on day 25, and the other four samples in the negative control group did not show any bacterial growth. Overall, 21 samples in the G120 group and 23 samples in the G200 group showed bacterial growth (Table 1). The median survival rate was 39.0 (25.0) days for the G120 group and 34.0 (25.0) days for the G200 group. Despite a slightly higher survival rate in the G120 group, the difference between the two experimental groups was not statistically significant (*p* = 0.612; Table 2, Figure 2).

**Table 1:** Frequency of bacterial colonization in different groups.

Group	Number	Number of events	Percentage	*p*‐value^a^
G120 (120°C)	41	21	51.2%	0.088
G200 (200°C)	41	23	56.1%	
Negative control	5	5	100%	
Positive control	5	1	20.0%	

a Fisher's exact test.

**Table 2:** Median survival time.

Group	Median	IQR	First quartile	Third quartile	Log rank *p*‐value
G120 (120°C)	39.0	25.0	4.0	40.0	0.612
G200 (200°C)	34.0	25.0	4.0	40.0	
Negative control	1.0	1.0	1.0	1.0	
Positive control	37	40	40.0	40.0	
Overall	37.5	25.0	1.0	40.0	

Abbreviation: IQR, interquartile range.

## 4 Discussion

The present results revealed that the two tested temperatures of the electrical heat carrier (120°C and 200°C) did not have any statistically significant effect on bacterial leakage of root canals obturated with the CWC technique using the Endoseal MTA sealer. Therefore, the null hypothesis is accepted.

Heating can significantly affect the properties and clinical performance of endodontic bioceramic sealers. High temperatures may cause thermal degradation of the organic components, which can alter the sealer's flowability, setting time, and film thickness (Antunes et al. 2021). Furthermore, heat can impact the solubility of bioceramic sealers; for instance, calcium silicate–based sealers tend to become more soluble when exposed to high temperatures, which might compromise their ability to maintain a seal in the root canal (Karam et al. 2024). Conversely, some endodontic bioceramic sealers are not significantly impacted by heating due to their inherent properties and composition (La Rosa et al. 2024; Ashkar et al. 2024).

To our knowledge, there is no previous study on the effect of temperature on the sealing ability of sealers and bacterial leakage using the CWC technique. In fact, all of the previous studies have focused on the physical–chemical properties of calcium silicate–based sealers affected by heat.

Jung et al. evaluated the effect of three different temperatures (100, 200, and 350°C) and different penetration depths of the System B plugger on root canal fillings (Jung et al. 2003). Although they found no significant difference among the three different temperature settings, their methodology was considerably different from the methodology of the present study; for example, they measured the percentage of the gutta–percha–filled area, and artificial oval canals were used in their study.

Yamauchi et al. assessed the effect of temperature on the physical properties of Endoseal MTA, and demonstrated that increasing the temperature decreased the setting time and flow rate of the sealer, but increased its film thickness (Yamauchi, Watanabe, and Okiji 2021). However, they did not assess the effect of temperature on the sealing ability of the sealer in actual root canals and its influence on bacterial leakage. In fact, such changes in the physical properties of sealers may not necessarily affect their sealing ability and bacterial leakage. Additionally, Atmeh and AlShwaimi investigated the effect of temperature on the chemical properties of sealers, including calcium silicate‐based sealers (Atmeh and AlShwaimi 2017). They showed that temperatures of 200 and 250°C had no significant effect on the chemical properties of calcium silicate‐based sealers.

Although bacterial leakage of the Endoseal MTA sealer was not affected by temperature in the present study, Yamuaichi et al. reported that some physical properties of this sealer changed following heat exposure (Yamauchi, Watanabe, and Okiji 2021). Also, it should be noted that in their study, the physical properties of the sealer were not evaluated in the root canals, and their methodology was different from that of the present study.

In summary, the changes in the physio‐chemical properties of calcium silicate‐based sealers observed in various laboratory studies can impede a clinician's ability to effectively manipulate these sealers during root canal filling procedures that involve heat in their obturation techniques. Poor manipulation, accelerated setting times, and reduced flow can result in inadequate distribution of the sealer, potentially compromising the quality of the root canal filling. This may lead to voids or gaps that harbor bacteria and allow microbial ingress, ultimately causing treatment failure or postoperative complications (Dahlen and Möller 1992). Therefore, theoretically, it seems prudent to evaluate root fillings for bacterial leakage after being subjected to a warm obturation technique, as we have done in the current study.

According to Timpawat et al. the use of a bacterial leakage model has greater clinical and biological relevance than the dye penetration technique for assessment of sealing ability (Timpawat, Amornchat, and Trisuwan 2001). However, bacterial studies are qualitative rather than quantitative, and leakage of even one bacterium can lead to turbidity of the medium due to its fast multiplication in the rich broth (Youngson et al. 1999; Chailertvanitkul et al. 1997). Nonetheless, it should be noted that different results may be obtained in the clinical setting due to the presence of body defense mechanisms (Jafari et al. 2017).

The long‐term stability of a sealer is another important factor that was not taken into account in the present study and should be addressed in future studies (De‐Deus et al. 2022). Moreover, the main confounder in leakage studies is the internal complex anatomy and dynamic environment of the actual root canal systems. If the experimental samples are not anatomically similar, the sample selection method fails to provide a consistent baseline for comparative assessments (De‐Deus et al. 2022).

It is recommended that future studies use a combination of different methods for evaluation of the quality of root canal fillings, for example, microcomputed tomography in conjunction with a bacterial leakage model. Moreover, long‐term follow‐ups can aid in achieving more accurate results.

## 5 Clinical Implication

The effect of heat on bioceramic sealers varies based on their composition. Some sealers are significantly affected by heat, whereas others remain stable (La Rosa et al. 2024). Clinicians should carefully choose the appropriate bioceramic sealer when using warm obturation techniques. If a bioceramic sealer is sensitive to heat, alternative filling techniques, such as cold‐ or heat‐modified obturations, should be considered (Motamedi, Gilbert, and Ha 2024). Integration of laboratory findings with clinical evidence is crucial for enhancing treatment efficacy and patient care.

## 6 Conclusion

According to the present results, the tested temperatures of the electrical heat carrier (120°C and 200°C) did not have a significant effect on bacterial leakage of root canals obturated by the CWC technique and the Endoseal MTA bioceramic sealer. The results indicated that both temperatures led to a similar level of bacterial leakage.

## Author Contributions

**Mir Ahmad Nabavi:** data curation, formal analysis, investigation, writing–review and editing. **Mahmood Reza Kalantar Motamedi:** conceptualization, methodology, writing–original draft, writing–review and editing. **Pedram Fattahi:** writing–original draft, writing–review and editing. **Saber Khazaei:** conceptualization, methodology, supervision, validation, visualization, writing–review and editing.

## Ethics Statement

The study protocol was approved by the regional bioethics committee of Kermanshah University of Medical Sciences (Approval No. IR.KUMS.REC.1400.837).

## Conflicts of Interest

The authors declare no conflicts of interest.

## Data Availability

The data that support the findings of this study are available from the corresponding author upon reasonable request.
